# Cross-reactive Antibodies against Avian Influenza Virus A (H5N1)

**DOI:** 10.3201/eid1509.090471

**Published:** 2009-09

**Authors:** Sathit Pichyangkul, Anan Jongkaewwattana, Arunee Thitithanyanont, Peeraya Ekchariyawat, Suwimon Wiboon-ut, Amporn Limsalakpetch, Kosol Yongvanitchit, Utaiwan Kum-Arb, Rangsini Mahanonda, Pongsak Utaisincharoen, Stitaya Sirisinha, Carl J. Mason, Mark M. Fukuda

**Affiliations:** Armed Forces Research Institute of Medical Sciences, Bangkok, Thailand (S. Pichyangkul, A. Limsalakpetch, K. Yongvanitchit, U. Kum-Arb, C.J. Mason, M.M. Fukuda); National Center of Genetic Engineering and Biotechnology, Bangkok (A. Jongkaewwattana); Mahidol University, Bangkok (A. Thitithanyanont, P. Ekchariyawat, S. Wiboon-ut, P. Utaisincharoen, S. Sirisinha); Chulalongkorn University, Bangkok (R. Mahanonda)

**Keywords:** Intravenous immunoglobulin, cross-reactive antibodies, H5N1 virus, influenza, viruses, letter

**To the Editor:** Intravenous immunoglobulin (IVIg) is used to treat bacterial and viral infections in patients with primary immunodeficiency disease and those with autoimmune and inflammatory disorders (*1*). IVIg contains pooled IgG from >1,000 blood donors and antibodies against various common human pathogens, including influenza virus A.

We tested the efficacy of commercial preparations of IVIg (50 mg/mL of highly purified immunoglobulin) against homosubtypic influenza viruses A (H1N1 and H3N2) and their cross-reactivity against avian influenza virus A (H5N1). IVIg preparations (Octagam; Octapharma, Vienna, Austria and Flebogamma; Instituto Grifols, Barcelona, Spain) had hemagglutination inhibition (HI) titers against subtypes H1N1 and H3N2 ranging from 20 to 40. Human Immunoglobulin, pH 4.0, (Harbin Sequel Bio-Engineering Pharmaceutical, Harbin, People’s Republic of China) had lower HI titers against homosubtypic avian influenza viruses (10 for subtype H3N2 and <10 for subtype H1N1). As expected, we did not detect antibodies against hemagglutinin (HA) of subtype H5N1 (A/open-billed/stork/Nahkonsawan/BBD0104F/2004) in any of the IVIg preparations (HI titer <10).

Human influenza subtype H1N1 shares the same neuraminidase (NA) subtype (human N1) as subtype H5N1 (avian N1). We therefore tested whether IVIg preparations would react and inhibit NA activity of human and avian influenza viruses by using a neuraminidase inhibition (NI) assay (*2*). NI titer was defined as the reciprocal of the highest dilution that gave 50% reduction compared with that of the virus control.

All 3 IVIg preparations inhibited NA activity of human N1 (NI titer against subtype H1N1 range 258–986) and human N2 (NI titer against subtype H3N2 range 1,309–3,274). Enzyme activity of avian N1 (7:1 reassortant; PR8 + NA [A/Vietnam/DT-0361/2005 H5N1]) was inhibited by all IVIg preparations (NI titer range 143–231). These findings support the recent observation of neutralizing antibodies against human N1 in human serum, which could inhibit enzyme activity of avian N1 from subtype H5N1 (*3*,*4*). We also tested IVIg preparations against reverse genetics subtype H5N3 virus in which the N3 NA was derived from H2N3 virus (6:1:1 reassortant; 6 internal genes from PR8 + HA (A/Vietnam/DT-0361/05 H5N1) + NA (A/duck/Germany 1207 H2N3) and observed no effect (NI titer <10). The N3 subtype belongs to avian influenza NA. Thus, antibodies against NA in IVIg appear to be specific for those circulating human influenza viruses (human N1 and human N2).

Unlike HA and NA, virus matrix 2 ectodomain (M2e) is highly conserved. Its presence on the surface of the viral particle makes it a potential target of antibody response similar to that for HA and NA (*5*,*6*). We assessed reactivity of IVIg preparations against a consensus M2e peptide derived from human influenza viruses of H1, H2, and H3 subtypes (MSLLTEVETPIRNEWGCRCNDSSD) and those derived from A/Hong Kong/156/97 H5N1 (MSLLTEVETLTRNGWGCRCSDSSD and A/Thailand/ SP-83/2004 H5N1 (MSLLTEVETPTRNEWECRCSDSSD) by using ELISA (*7*). Antibody titer was defined as the reciprocal of the highest dilution that had an optical density of 0.5 at 414 nm in our assay.

Results showed considerable variation among IVIg preparations, caused by M2e peptides derived from different influenza viruses (titer range 88–23,614). Among the 3 preparations, Human Immunoglobulin, pH 4.0, IVIg showed the highest titers against all M2e peptides (consensus, 9,639; H5N1 Hong Kong, 3,519; and H5N1 Thailand, 23,614). Variation of antibody titers against M2e in IVIGs may be geographically dependent. Unlike Octagam and Flebogamma, Human Immunoglobulin, pH 4.0, IVIg was likely derived from blood donors in China. Octagam and Immunoglobulin, pH 4.0, IVIg were more reactive with M2e of avian influenza virus (H5N1) (A/Thailand/SP-83/2004) than with other M2e peptides.

We measured the ability of IVIg preparations to inhibit influenza subtype H5N1 replication by using a plaque-reduction assay. Subtype H5N1 (A/open-billed stork/ Nakhonsawan/BBD0104F/2004) was maintained as described (*8*). MDCK cells were infected with virus and agar containing various concentrations of IVIg was layered on top of these cells and incubated for 2 days. Results are shown in the Figure. IVIG inhibited plaque formation in a dose-dependent manner. Although plaques of heterogeneous size were observed in infected plates without IVIg, larger plaques were preferentially neutralized with increasing concentrations of IVIg in the agar (Figure).

**Figure:**
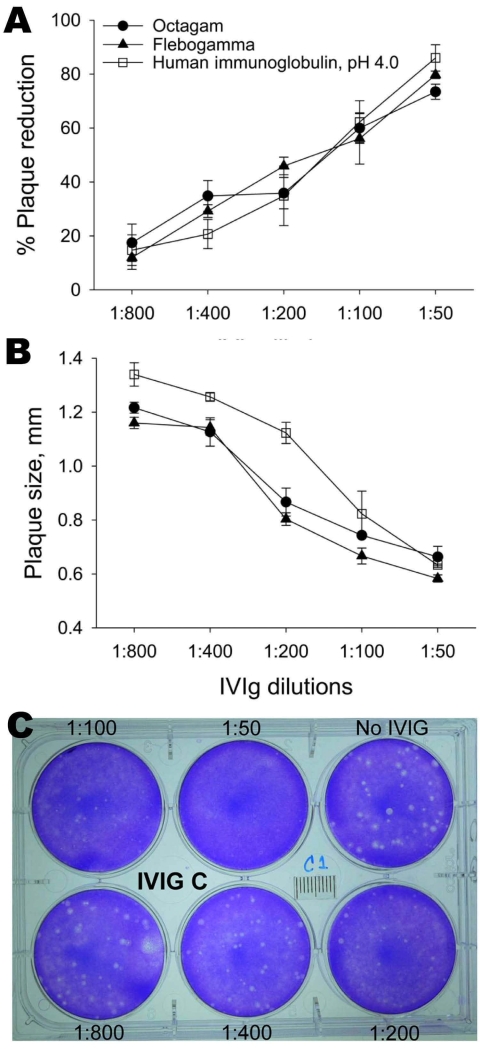
Neutralization of avian influenza virus A (H5N1) by intravenous immunoglobulin (IVIg) preparations measured by percentage reduction in plaque number (A) and plaque size (B). Monolayers of MDCK cells were infected with virus and overlaid with agar containing various concentrations of IVIg. After 2 days, plaques were detected by staining with crystal violet. Shown is a sample of viral plaques with agar overlay containing different dilutions (1:50–1:800) of Human Immunoglobulin, pH 4.0, (Harbin Sequel Bio-Engineering Pharmaceutical, Harbin, People’s Republic of China) IVIg (C). Data are mean ± SE of 3 experiments.

Premixing excess M2e peptide with IVIg to absorb M2e-specific antibodies had no effect on plaque formation, indicating that antibodies against M2e in IVIg preparations were not responsible for neutralization of influenza subtype H5N1. Antibodies against M2e may have a role in protection against subtype H5N1 by another mechanism.

Our data suggest that the neutralizing activity against influenza subtype H5N1 in all 3 IVIg preparations was likely contributed by cross-reactive antibodies against avian N1. IVIg has been reported to have antiinflammatory activity (*9*,*10*). The immune suppressive effect of IVIg may benefit patients by reducing the cytokine storm. These data suggest use of IVIg, especially preparations containing high neutralizing activity against subtype H5N1, as adjunctive treatment for infection with highly pathogenic avian influenza virus (H5N1).
